# Implementation of a Design of Experiments to Improve Periplasmic Yield of Functional ScFv Antibodies in a Phage Display Platform

**DOI:** 10.34172/apb.2022.061

**Published:** 2021-07-03

**Authors:** Marjan Abri Aghdam, Mohammad Reza Tohidkia, Elham Ghamghami, Asadollah Ahmadikhah, Morteza Khanmahamadi, Behzad Baradaran, Ahad Mokhtarzadeh

**Affiliations:** ^1^Department of Biological Science, Faculty of Basic Science, Higher Education Institute of Rab-Rashid, Tabriz, Iran.; ^2^Research Center for Pharmaceutical Nanotechnology, Tabriz University of Medical Sciences, Tabriz, Iran.; ^3^Faculty of Life Sciences and Biotechnology, Shahid Beheshti University, G.C Velenjak, Tehran, Iran.; ^4^Chemical Engineering Faculty, Sahand University of Technology, Sahand New Town, Tabriz, Iran.; ^5^Immunology Research Center, Tabriz University of Medical Sciences, Tabriz, Iran.

**Keywords:** Single-chain variable fragment (scFv), Phage display, Response surface methodology, Periplasmic expression, Optimization, D-optimal design

## Abstract

***Purpose:*** Production of functional recombinant antibody fragments in the periplasm of *E. coli* is a prerequisite step to achieve sufficient reagent for preclinical studies. Thus, the cost-effective and lab-scale production of antibody fragments demands the optimization of culture conditions.

***Methods:*** The culture conditions such as temperature, optical density (OD_600_) at induction, induction time, and IPTG concentration were investigated to optimize the functional expression of a phage-derived scFv molecule using a design of experiment (DoE). Additionally, the effects of different culture media and osmolyte supplements on the expression yield of scFv were examined.

***Results:*** The developed 2FI regression model indicated the significant linear effect of the incubation temperature, the induction time, and the induction OD_600_ on the expression yield of functional scFv. Besides, the statistical analysis indicated that two significant interactions of the temperature/induction time and the temperature/induction OD_600_ significantly interplay to increase the yield. Further optimization showed that the expression level of functional scFv was the most optimal when the cultivation was undertaken either in the TB medium or in the presence of media supplements of 0.5 M sorbitol or 100 mM glycine betaine.

***Conclusion:*** In the present study, for the first time, we successfully implemented DoE to comprehensively optimize the culture conditions for the expression of scFv molecules in a phage antibody display setting, where scFv molecules can be isolated from a tailor-made phage antibody library known as "Human Single Fold scFv Library I."

## Introduction

Antibodies are considered as valuable molecules for basic research, diagnosis, and therapeutic applications. However, the widespread use of antibodies as therapeutics has been hampered by production costs, stability, and allergic reactions.^1,2^ Recombinant DNA techniques have provided a perfect solution for the cloning and expression of antibody fragments as a promising platform for the production of therapeutic agents. The single-chain fragment variable (scFv), as the most commonly used antibody fragments and one of the smallest and functional forms of a conventional whole antibody molecule, is composite of the variable regions of light (V_L_) and heavy (V_H_) chains connected via a short flexible peptide. Fully human scFv antibodies lacking the constant Fc domain have been produced mainly through a phage display screening platform to circumvent some shortcomings of traditional full-length antibodies.^3-5^ The merit of *E. coli as a* prokaryotic host for producing non-glycosylated antibody fragments such as scFvs is mainly due to its fast growth, cost-effectiveness, and easy genetic manipulation.^6,7^ In contrast to the reducing condition in the cytoplasm, which strongly counteracts the formation of disulfide bonds, the oxidizing environment in the bacterial periplasm provides the formation of disulfide bonds through periplasmic foldases and disulfide isomerase. The periplasm also contains fewer proteases which minimize proteolytic degradation compared to the cytoplasm and accounts for only 4-8% of the *E. coli* host cell proteins.^8,9^ Moreover, periplasmic space offers selective extraction approaches that preserve the inner membrane while destabilizing the outer membrane and thereby, fewer purification efforts are needed due to the lower host proteins contamination.^10,11^

The periplasmic expression system has frequently been used as a preferred strategy downstream of combinatorial phage display library screening to produce an adequate amount of a soluble scFv for experimental and preclinical characterizations such as specificity, binding affinity, and functional assays *in vivo* and *in vitro.*^12^ However, the high-level expression of antibody fragments with a signal peptide in the periplasm space is often accompanied by the formation of biologically inactive protein aggregates or inclusion bodies after transport to the periplasm.^13^ Therefore, optimization of culture conditions is a critical step to achieve a high level of soluble and functional scFv fragments. In most protein expression studies, the optimization is routinely obtained by altering just one parameter at a time while maintainingthe others constant. Such a method requires labor-intensive experiment runs without the capability to represent the interaction between variable.^14,15^ and can cause misinterpretation of the results due to potential interactions between different variables.^16^ To overcome these limitations, the best approach is using the statistical design of experiments (DoE) methodology to simultaneously evaluate the linear effects of different variables and their interactions on one or more responses.

However, to our knowledge, there is no report onthe extensive andsystematic optimization of the cultivation conditions for the lab-scaleexpression of soluble scFv fragments using a phagemid vector during phage display trajectories. To address this, in the present study, we initially applied DoE to optimize culture and induction conditions for functional expression of an scFv in *E. coli* by selecting four variables, including temperature, optical density (OD_600_) at induction, Isopropyl β-D-1-thiogalactopyranoside (IPTG) concentration, and induction time. The flow diagram of the procedure applied in this study is illustrated in Figure S1 (See Supplementary file 1). Moreover, to further improve optimal expression yield, we investigated the effects of different types of culture media (i.e., TB, LB, phosphate-buffered LB, 2xYT, and phosphate-buffered 2xYT) and various medium additives (i.e., 0.4 M sucrose, 100 mM glycine betaine, 0.5 M sorbitol, 0.05% glycerol, and 4% NaCl) on the expression level of functional anti-G17-Gly scFv at optimal culture condition.

## Materials and Methods

### Bacterial strains and plasmids

The recombinant anti-G17-Gly scFv was previously isolated from a semi-synthetic phage-scFv library (Tomlinson I Library) based on the randomized human single framework for VL (DPK9 and Jκ1) and VH (V3-23 and JH4b) chains against peptide hormone G17-Gly.^17^ The given antibody clone, which was transformed toan amber non-suppressor *E. coli* strainHB2151 (provided by the library), was located in the phagemid vector pIT2 harboring an ampicillin-resistant marker, the *pelB* signal sequence for periplasmic expression, an IPTG-inducible lac promoter, and His and C-myc tags for characterization.

### Periplasmic scFv expression and extraction

The antibody clone containing the pIT2/anti-G17-Gly scFv vector was cultured overnight at 37°C on a TYE plate supplemented with 100 μg/mL ampicillin and 2% glucose. The overnight culture was prepared by inoculation of 5mL 2xYT medium containing 100 μg/mL ampicillin and 2% glucose with a single colony from the TYE plate and incubation at 37°C for 16 hours under shaking at 160 rpm. The next day, the culture was diluted at a ratio of 1:100 with 15 ml of fresh 2xYT medium and grown at 37 °C until the certain OD_600_ at induction (Table S1). According to the experimental design, varied induction conditions consisting of all potential combinations of the four variables(OD_600_ at induction, induction time, IPTG concentration, and temperature) in different levelswere performed.To normalize the cell density for each sample,the bacteria cells from[15/OD600] mL of culture were pelleted by centrifugation for 10 minutes at 4500 rpm (at 4*°*C)and then resuspended gently in 1 mL of ice-cold TES (50 mM Tris pH 7.2, 0.5 mM EDTA, and 20 % sucrose)/lysozyme (1 mg/mL) buffer.^18^ After a 30 minutes incubation on ice, the cell suspension was centrifuged at 16000 rpm for 30 minutes at 4°C to obtain total periplasmic extraction. The supernatant containing soluble scFvs were collected and kept at -20°C until further analysis for the expression of functional soluble scFv by ELISA assessment.

### Design of experiments and statistical analysis

Response surface methodology (RSM) based on D-optimal design was used to statistically investigate the effect of four independent variables, including temperature, OD_600_ at induction, induction time, and IPTG concentration on the functional production anti-G17-Gly scFv in *E. coli* HB2151. Experimental Data analysis was undertaken by using Design-Expert® software version 7 (Stat-Ease, Inc. Minneapolis). After data collection, they were examined for normality through a normal probability plot to ensure eligibility for statistical analysis. Afterward, the model generated were checked for statistical significance with the help of STATISTICA 9. The significance of individual variables and related interaction effects was determined using variance (ANOVA) analysis, and the statistically significant effects were considered where the probability value was less than 0.05 (*P* ˂ 0.05).

### ELISA assay

An indirect antigen-coating ELISA assay was applied to evaluate the concentration of the functional anti-G17-Gly scFv expressed in different conditions. Concisely, 96-well microtiter plates (Biomat) were coated with 100 µL/well of biotinylated-BSA (Thermo Fisher Scientific, Waltham, MA) at a concentration of 2 µg/mL in phosphate-buffered saline (PBS) overnight. The plates were washed three times with PBS containing 1% Tween-20 (v/v, PBST) and then incubated with 100 µL/well streptavidin (Bio Basic) at a concentration of 10 µg/mL in PBS at room temperature while shaking gentle. Following a 90-min incubation and washing step, the plates were coated with 100 µL/well of the 200 nM biotinylated peptide (pEGPWLEEEE-K-s-s-biotin, pE denotes pyroglutamic acid) at room temperature for 90 minutes with gentle shaking on a shaker. The plates were blocked with 2% MPBS (skimmed milk powder in PBS, w/v) and incubated with the scFv samples diluted in 2 % MPBS at room temperature for 90 min. The plates were incubated with protein L-HRP (Thermo Fisher Scientific), recognizing the kappa light chain of scFv molecules, at a dilution of 1:5000 for 1 hour to detect the specific binding of anti-G17-Gly scFv. After a washing step, the visualization was carried out by adding 100 µL TMB substrate (of 3, 3′, 5, 5′-tetramethylbenzidine) per well. The enzyme reaction was halted using 50 µL of 5% sulfuric acid, and then OD was read at 450 nm subtracted from 630 nm (as background wavelength) using an ELISA plate reader (BioTek, Winooski, VT, USA). The immobilized metal affinity chromatography (IMAC)-purified anti-G17-Gly scFv with a specified concentration was used to establish a standard curve for concentration calculation of unknown test samples.^5^ Test samples were diluted 1:10 in MPBS to reduce oversaturated signals due to the high concentration of the scFv. Negative control samples without scFv content were also included in the ELISA assays to subtract background signals.

## Results and Discussion

### Analysis of experimental design and modeling

The current study aimed to predict and develop a unified approach for the expression of functional scFv antibody fragments obtained from the human single-fold scFv Tomlinson I library in *E. coli* HB2151 and shaking flask. To this end, four major variables, including temperature (18 to 30°C), IPTG concentration (0.05 to1 mM), induction time (4 to 24 hours), and OD_600_ at induction (between 0.5 and 1.5) were selected to study their effects on the expression of anti-G17-Gly scFv (Table S1). Instead of the ineffective and time-consuming conventional method, which works by changing the level of one variable at a time while holding certain variables constant, DoE was applied here because it enables to determine the linear influence of several parameters, the possible interactions between variables, and the optimum combination of all the variables within one set of experiments. Moreover, herein, ELISA assay was used to measure the expression yield of the merely functional, soluble fraction of scFv molecules, rather than widely used SDS-PAGE analysis, which measures total yield of the soluble fraction of both functional and nonfunctional scFv molecules. The ELISA test is a reliable and sensitive assessment since it displays the binding properties of appropriately folded scFv fragments (correctly folded disulfide bonds) at the periplasmic space, excluding soluble aggregates, unfolded, or misfolded scFv variants from the analysis.^19,20^

Accordingly, 80 experimental conditions wereconducted using RSM and D-optimal design to examine the expression level of functional soluble scFv by ELISA. The total volumetric production (mg/L) of thescFv was in the range of 0.037-16.6 mg/L under different culture conditions, indicating the great importance of defining an optimal culture condition.

**Figure 1:**
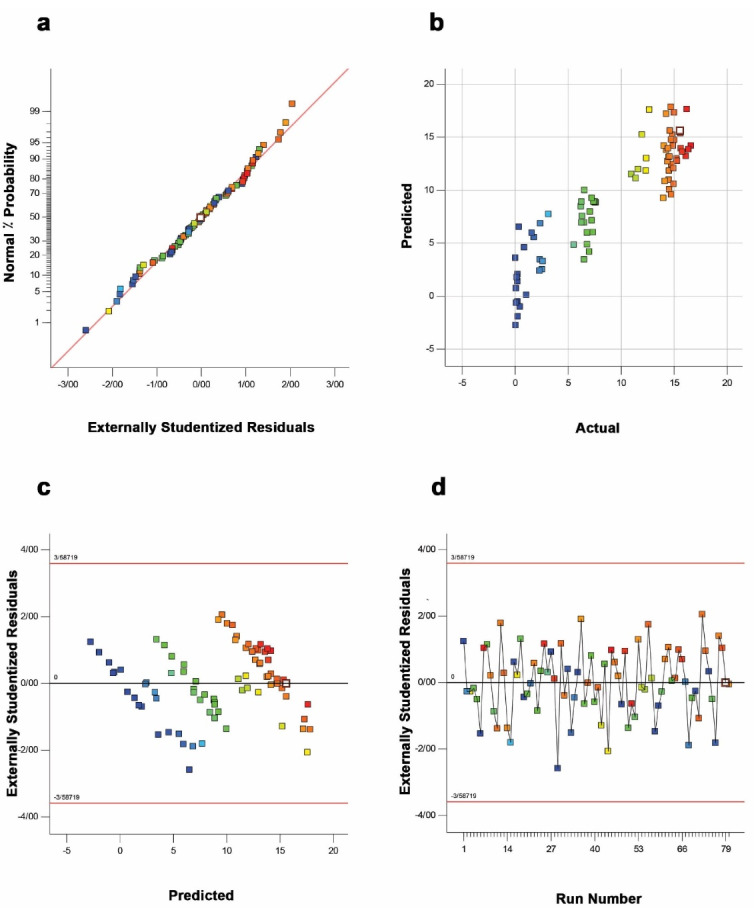
The authorization of the 2FI regression model by the studentized residual plots. a) The normal plot of residuals; b) the predicted vs. actual plot; c) the residuals vs. predicted plot, and d) the residuals vs. run number plot.

Based on the adopted DoE, among different plausible models, the response surface 2FI regression model was identified as a suitable model to explore the effects of variables on the total volumetric production.A variety of statistical tests realized the significant adequacy of the 2FI model. For instance, the high value of correlation coefficient (R^2^ = 0.9512) for the model and the satisfactory agreement of “Pred R-Squared” (0.8666) with the “Adj R-Squared” value of 0.9197 revealed a high correlation accuracy between the predicted and actual values. Adeq Precision” with a high value of 16.99 also indicated a satisfactory ratio of signal to noise. Alternatively, the studentized residuals provide a robust criterion for identifying outliers and estimation of regression model function. As shown in Figure 1a, the plot of normal probability for the scFv production was appeared as a linear distribution of the points, demonstrating no deviation from the variance. While the plot of predicted values (software estimated) versus actual values (experimental calculated) indicated that the model could predict the response accurately (Figure 1b), the plot of residuals versus the respective predicted values demonstrated a desirable regression model upon randomly distributed data (Figure 1c). The plot of residuals versus the experimental run lies within the limits as given in Figure 1d. Overall, these statistical analyses revealed that all data points follow a normal distribution pattern, and the model can be used to predict the response. Additionally, the statistical significance of the 2FI model was also established by the ANOVA analysis and F-test. As shown in Table 1, the F-value of 30.2 with a low probability value (*P* ˂ 0.0001) and a non-significant lack of fit F-value (1.48, *P* ˃ 0.05) indicated that the model terms were significant and there is only a 0.01% chance that this F-Value might happen owing to noise.

**Table 1:** ANOVA results for Response Surface 2FI Model.

**Source**	**Sum of Squares**	**df**	**Mean Square**	**F Value**	***P*** ** value Prob > F**	
Model	2547.51	31	82.18	30.20	˂0.0001	Significant
A-IPTG	7.97	1	7.97	2.93	0.0935	
B-OD_600_	101.26	1	101.26	37.21	< 0.0001	Significant
C-Induction Time	425.12	4	106.28	39.06	˂0.0001	Significant
D-Temperature	1615.04	2	807.52	296.78	< 0.0001	Significant
AB	1.70	1	1.70	0.63	0.4331	
AC	15.23	4	3.81	1.40	0.2484	
AD	1.10	2	0.55	0.2	0.8170	
BC	16.70	4	4.18	1.53	0.2071	
BD	19.02	2	9.51	3.50	0.0383	Significant
CD	255.92	8	31.99	11.76	0.0001	Significant
A^2^	4.85	1	4.85	1.78	0.1883	
B^2^	2.48	1	2.48	0.91	0.3446	
Lack of Fit	121.09	43	2.82	1.48	0.3557	Not Significant
Residual	130.61	48	0.13			
Pure Error	9.51	5	1.90			
Cor Total	2678.12	79				

### Effects of culture conditions on the scFv expression

**Figure 2:**
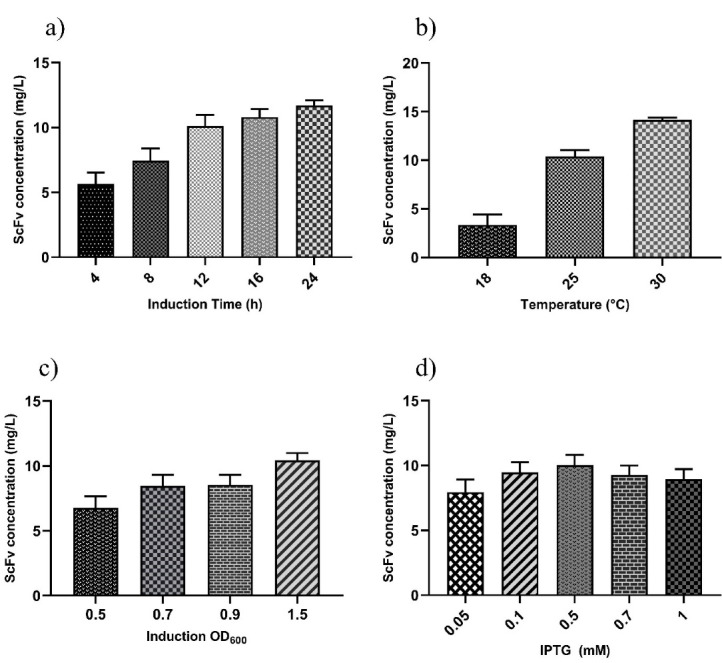
The linear functions of variables. The individual effects of four independent variables, including induction time (a), temperature (b), OD_600_ at induction (c), and IPTG concentration (d), on the expression yield of scFv were evaluated.

The ANOVA analysis revealed that the linear terms of induction OD_600_ (B), induction time (C), and temperature (D) as well as the interaction terms of induction time/temperature (CD) and induction OD_600_/temperature (BD), had a significant positive influence on the functional scFv expression (*P* < 0.05, Table 1). The main effect of each variable without considering the relationship among the 4 variables showed that statistically significant linear terms, including induction time, the temperature, and induction OD_600_ had a positive effect on scFv production yield (*P* ˂ 0.0001). Long induction time can significantly promote the functional expression of scFv in *E. coli* periplasm. By screening various induction times (i.e., 4, 8, 12, 16, and 24 hours), the highest and lowest yield of functional scFv were obtained 24 *P* and 4 *P* after IPTG induction, respectively (11.7 vs. 5.6 mg/L) (Figure 2a). Similarly, the functional yield of scFv enhanced significantly through an increase in the incubation temperature from 18°C to 30°C(Figure 2b) as well as through increasing the induction OD_600_ from 0.5 to 1.5 (Figure 2c).The ANOVA results also showed that induction OD_600,_ as a linear term, had a positive impact on the functional yield of scFv, indicating that the G17-Gly scFv expression yield increased with higher OD values. It means that an additional increase in the cell density before induction through using baffled shake flasks has the potential benefit to increase the scFv yield further. In line with this observation, Kasli and coworkers showed that raising cell density from 0.62 to 1.21 improved productivity yield of pelB^sp^-scFv in induction OD_600_ perturbation curve, but with negligible effect on solubility, demonstrating high density of cell factory at the beginning of induction can improved scFv yield without compromising its solubility.^21^ However,IPTG concentration (A) in the range of 0.05-1 mM was considered as a non-significant linear termbecause increasing the concentration of IPTG did not produce an increasing trend in the scFv yield (Figure 2d).

**Figure 3:**
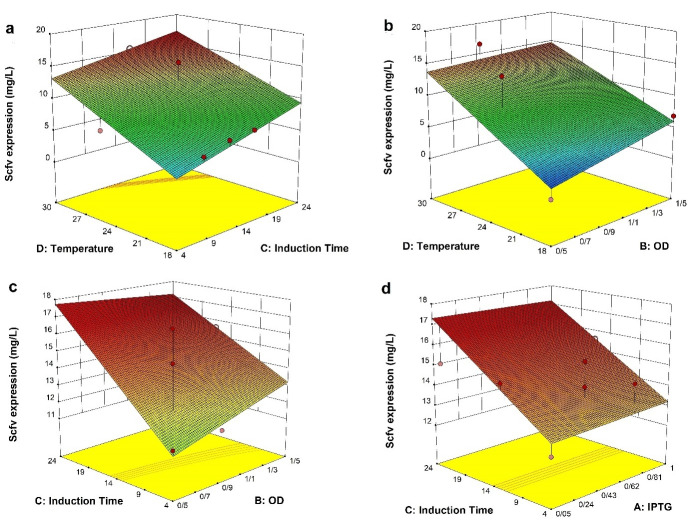
The interaction functions of variables. The response surface plots represent the interaction effects of a different variable on the expression of scFv. The blue color signifies the minimum value (0.03 mg/L), whereas the red color represents the maximum value (16.6 mg/L). a) The induction time/temperature time interaction (CD), b) The induction OD_600_/temperature interaction (BD), c) The induction OD_600_/induction time interaction (BC), d) The IPTG/induction time interaction (AC).

The interactive effects of independent variables were illustrated by plotting 3D response surfaces (Figure 3). Temperature and induction time strongly interacted to increase the expression yield (CD, *P* = 0.0001); i.e., when a high cultivation temperature was coupled with a high induction time (Figure 3a), total volumetric productivity exceeded 15.59 mg/L. Perhaps, both increased bacterial density and transcription and translation rate at relatively high temperatures (30*°*C) can contribute to this high yield. The positive effect of temperature was similarly reported by Kasli et al^21^ who found that the production yield and the solubility of pelB^sp^-scFv (with secretory Sec pathway signal peptide) were amplified with increasing temperature from 20°C to 30°C. Also, in agreement with our findings, Larentis et al^22^ reported that the expression of mature rPsaA in *E. coli* BL21 (DE3) star was higher at 25*°*C once the incubation time extended for 16 hours after the induction, confirming the interaction between these two variables. Controversially, several studies promote solubility, folding, and resultant functional yield of scFvs by stress minimizing through either adopting lower growth temperatures or low inducer concentrations to permit balancing between bacterial growth and recombinant protein production. This discrepancy with our results could be explained by differences in the origin of the scFv molecule, primary sequence, expression vector, and host strain. It was frequently found that scFvs derived from combinatorial phage antibody libraries become more soluble than mouse-derived scFv molecules because phage-displayed scFv antibodies are selected for both affinity binding and solubility.^23^ Additionally, some combinatorial phage antibody libraries (e.g., HuCAL and Tomlinson I + J libraries) have been constructed by grafting natural or randomized CDR regions on a single or multiple gremlin framework scaffolds, which was identified by exploiting directed evolution approaches, to assist for the display and expression of functional antibody fragments with appropriate solubility and stability properties.^24,25^ In this respect, the current study utilized the anti-G17-Gly scFv isolated from a tailor-made phage antibody library named “Tomlinson library I”, which are based on a well-expressed single human framework VH-VL (V3-23/DPK9) and prescreened for the binding to both protein L and protein A so that most of the clones presented by the unselected library become functional. Lastly, it was previously stated that in contrast to stronger tac or T7/lac promoters, an expression vector containing moderate lac promoter provides a less intracellular accumulation of scFv and a more soluble periplasmic fraction.^23^ Accordingly, as opposed to most studies that utilized specialized materials such as vectors based on robust T7/lac promoter (e.g., pET derivatives) and BL21 (D3) as a host to evaluate effects of different culture conditions on scFv expression.^26-28^ Here, in a phage display setting, we used a phagemid containing lac promoter with moderate strength and HB2151 strain that allow effective balancing between cell growth and recombinant protein synthesis, thereby preventing metabolic load through long-term incubation at 30*°*C.

In addition, a statistically significant interaction but not firm was also found between temperature and OD_600_ (BD, *P* = 0.038) in a way that the increase of OD value at a higher temperature produced fair improvement in the scFv yield (Figure 3b). Similar results were also reported that induction of recombinant proteins at late growth phase in some bacterial strains is favorable over that in early log-phase, where recombinant host engages the whole cellular machinery and carbon/energy source for the expression of recombinant protein, and thereby making slower cell proliferation and lower cell densities.^29,30^

The surface response of interaction between induction time and OD_600_ at induction (BC, *P* = 0.2) displays a very slight, but no statistically significant, increase in the yield of functional scFv at early induction coupled with longer induction time (Figure 3c). A similar correlation, albeit in the different culture settings, was also observed for the expression of periplasmic scFv (in the baffled shake flasks) and Fab (in the fed-batch fermentation) whose production yield was the highest at 24 hours post-induction when the induction was done at a low biomass concentration.^21,31^ Though, the potential for inconsistency in the periplasmic scFv concentration and thereby stochastic expression increases in cultures induced at low biomass. Likewise, the ANOVA and response surface results showed that the interaction terms of induction time and IPTG concentration had not statistically significant impact (AC, *P* = 0.24) on the expression of soluble anti-G17-Gly scFv (Figure 3d). This IPTG concentration-independent effect was previously reported by Kipriyanov and colleagues, who demonstrated that the yield of soluble periplasmic scFv expressed under the control of the weaker lac promoter was not affected by different IPTG concentrations.^23^

### Optimum culture conditions

To interrogate the optimum culture conditions for producing the highest functional and soluble scFv in shaking culture flasks, 80 experimental runs were established by RSM and D-optimal. First, the most favorable culture conditions over each incubation time were screened based on volume productivity and displayed in Table 2. While the lowest volume productivity was displayed by 4 hours (14.57 mg/L), the highest volume productivity was achieved by incubation times of 12 hours and 24 hours (16.6 and 16.36 mg/L), in sequence, at specified culture conditions. On the other hand, the cost-effective production resulted from incubation time of 16 hours (induction at 0.1 mM IPTG) and 8 hours (induction at 0.5 mM IPTG), both with volume productivity of 15.6 mg/L. The maximum specific production, as a function of bacterial cells for the production of recombinant proteins, was achieved by incubation time of 12 h at the defined conditions (452.7 µg/mg total protein).

**Table 2:** Optimum culture condition for each induction time

**Induction time (h)**	**Temperature (°C)**	**Induction at OD**_600_	**IPTG (mM)**	**Volume productivity mg/L** ^a^	**Specific production µg/mg** ^b^
4	30	1.5	0.7	14.57	174.6
8	30	1.5	0.5	15.65	159.7
12	30	0.7	1	16.6	452.7
16	30	0.9	0.1	15.62	275.9
24	25	1.5	0.7	16.36	258.8

^a^Calculated based on ELISA assay and normalized to one liter of culture. ^b^Calculated by measuring the scFv concentration (determined by ELISA) per milligram of total protein fraction.

Based on the developed 2FI model through design-expert software, the optimum culture conditions were predicted as follows: the temperature of 25°C, 0.98 mM IPTG, induction time of 24 h, and induction at OD_600_ of 1.46. Themaximum yield of functional scFv under the optimum conditionspredicted by the regression model was found to be 17.19 mg/L. The validation test under the same optimum conditions enabled the production of scFv with a concentration of 17.35 mg/L, which was in close agreement with the model predicted yield of scFv. In contrast to the standard expression conditions (i.e., the temperature of 30°C, 1 mM IPTG, induction time of 4 h, and induction at OD_600_ of 0.9)with the yield of 11.6 mg/L, the validation test also exhibited a significant increase in the volume productivity (about 150 percent).

### Effects of the culture medium type and medium additives

**Figure 4:**
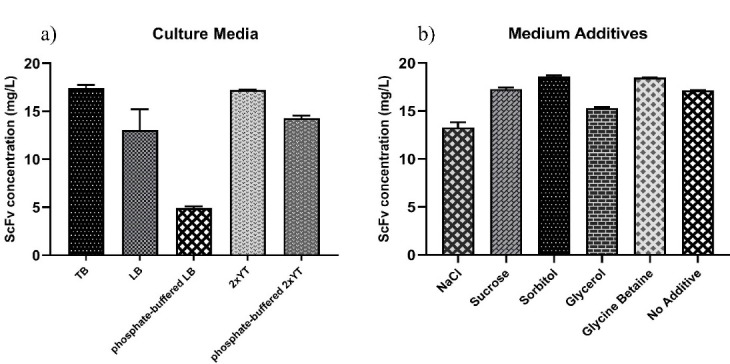
Effect of different culture media and medium additives on the scFv expression level. The expression performance of anti-G17-Gly scFv under the optimal culture conditions was analyzed through a) different culture media (TB, LB, PBS-LB, 2xYT, and PBS-2xYT); and b) medium supplements (4% NaCl, 0.4 M sucrose, 0.5 M sorbitol, 0.05% glycerol, and 100 mM glycine betaine). The 2xYT and no additive (2xYT with no supplements) were used as controls for culture media and medium supplement experiments, respectively.

To further improve the functional yield of scFv, we also screened the effect of different culture media and medium additives at optimum culture conditions.Different culture media (TB, LB, phosphate-buffered LB, 2xYT, and phosphate-buffered 2xYT) and well-known medium additives (0.4 M sucrose, 100 mM glycine betaine, 0.5 M sorbitol, 0.05% glycerol, and 4% NaCl) were analyzed for the augmentation of soluble/functional scFv expression by ELISA. As shown in Figure 4a, expression in bothTB and 2xYT media resulted in the maximum yield of scFv (17.41 and 17.02 mg/L, respectively) compared to other culture media, i.e., phosphate-buffered 2xYT, LB, phosphate-buffered LB. Likewise, in our previous study, TB was also found as a superior medium for the expression of four scFv clones in a microtiter plate scale to screen individual-specific binders from Tomlinson I + J libraries.^32^ This could be due to the presence of a carbon source (glycerol) and the additional amount of yeast extract in the composition of TB medium that supports the further growth of bacteria in high cell density. Besides, the expression yield of scFv cultured in the 2xYT and LB media was higher than the corresponding phosphate-buffered media, indicating that the expression of scFv was compromised by an extra amount of phosphate salt in the 2xYT and LB media.

It has been well-documented that the growth and induction of bacteria cells in the presence of osmotic stress (NaCl) and/or chemical osmolyte supplements (sorbitol, glycine betaine, glycerol, sucrose, L-arginine, formamide, acetamide, and ethanol) can lead to an increase in the level of soluble and functional scFv fraction via a combination of following ways: (a) induction of the intracellular network of molecular chaperones which assist refolding of unfolded proteins and prevent protein aggregation, (b) direct interaction of chemical osmolytes with recombinant protein to stabilize its native structure (chaperon activity), and (c) increasing protein solubility.^33^ The use of chemical osmolyte-assisted stress conditions in our hands resulted in a positive effect on the production of functional anti-G17-Gly scFv by the addition of 0.5 M sorbitol or 100 mM glycine betaine (18.61 mg/L and 18.48 mg/L scFv yield, respectively). In contrast, an opposite effect was observed when using 4% NaCl and 0.05% glycerol, due to, in some parts, having a detrimental influence on cell integrity at longer incubation time (24 hours) and high cell density (Figure 4b). Sucrose, which has been frequently utilized as a suitable osmolyte,^23,34^ particularly for the expression of bispecific antibody fragments,^35^ did not improve the yield of functional scFv in this study. The expression yield decreased to a level lower than non-additive medium control. This finding is in a rough consensus with a study conducted by Sandee et al., who reported that the soluble/insoluble ratio of Hep27 scFv was significantly increased by the 0.5 M sorbitol supplementation while adding NaCl, sucrose, and glycine betaine resulted in no improvement or even negative effect on the solubility scFv.^36,37^ Additionally, there are a couple of studies in which the consistent results were reported on the useless effect of 0.4 M sucrose and the negative effect of glycerol in enhancing soluble and function yield of scFvs in microplate platform expression.^36,38,39^

## Conclusion

The optimization of the recombinant protein expression is paramount to ensuring the productivity of high protein yields. However, the optimization process can be very consuming. By using DoE, the process optimization becomes more informative and more reliable. In the present study, DoE methodology was successfully used to optimize the culture conditions for the periplasmic expression of functional scFv in a phage display setting, where the expression of the antibody fragments isolated from any phage antibody library is a critical step to prepare a sufficient soluble and biologically active reagent for preclinical characterization such as specificity, binding affinity, and functionality *in vitro* and *in vivo*. The results demonstrated that temperature, post-induction time, OD_600_ at induction exhibit significant influence on the expression of scFv antibody fragment. The adapted model here proposed temperature of 25°C, 0.98 mM IPTG, post-induction time of 24 hours, and OD of induction 1.46 asthe optimal culture condition for the expression of scFv binders screened from “Human Single Fold scFv Library I.” Taken together, the methods optimized herein might contribute to the production of satisfactory amounts of scFv molecules for conducting functional studies on the biological role of these binders derived from tailored-made phage antibody repertoires, in particular,Tomlinson I + J libraries.

## Acknowledgments

The authors are very grateful for the technical support provided by the Immunology Research Center and the Research Center for Pharmaceutical Nanotechnology at Tabriz University of Medical Sciences.

This study was supported by the Immunology Research Center, Tabriz University of Medical Sciences, Tabriz, Iran (Grant number 60441).

## Ethical Issues

This article does not contain any studies with human participants or animals performed by any of the authors.

## Conflict of Interest

The authors have no conflicts of interest.

## Supplementary Materials


Supplementary file 1 contains Table S1 and Figure S1.
