# Supplementary material for: Implementation of a Design of Experiments to Improve Periplasmic Yield of Functional ScFv Antibodies in a Phage Display Platform
Source: Adv Pharm Bull. 2021 Jul 3;12(3):583–92. doi: 10.34172/apb.2022.061 (PMC9348535; doi:10.34172/apb.2022.061)
Supplement: Supplementary file 1 — contains Table S1 and Figure S1. [file apb-12-583-s001.pdf]

Table S1. The levels and variables in the D-optimal design experiments

Symbols	Variables					
A	IPTG concentration (mM)	0.05	0.1	0.5	0.7	1
B	OD_{600} at induction	0.5	0.7	0.9	1.5	
C	Induction time (h)	4	8	12	16	24
D	Temperature (°C)	18	25	30		

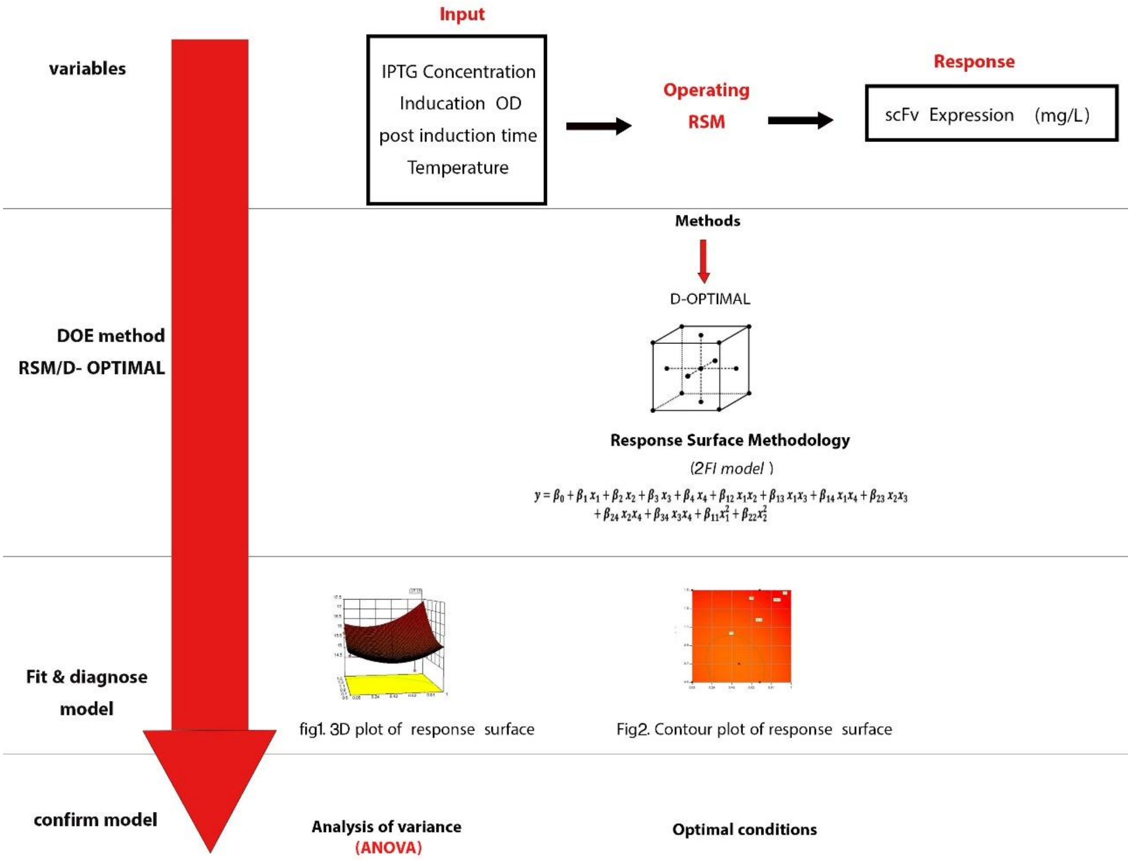

Figure S1. Schematic illustration of the methodology applied in RSM analysis. The sequential stages include the screening of independent variables, selection of the experimental design, implementation of experiments to obtain results, evaluation of the fitted model, confirming the significance of the model using analysis of variance (ANOVA), and determination of the optimum conditions.
